# Assessment of residual plant DNA in bulk milk for Grana Padano PDO production by a metabarcoding approach

**DOI:** 10.1371/journal.pone.0289108

**Published:** 2023-07-25

**Authors:** Anna Pozzi, Nelson Nazzicari, Rossana Capoferri, Slobodanka Radovic, Graziella Bongioni

**Affiliations:** 1 Istituto Sperimentale Lazzaro Spallanzani, Localita’ La Quercia, Rivolta d’Adda (CR), Italy; 2 CREA—Council for Agricultural Research and Analysis of Agricultural Economics, Research Centre for Animal Production and Aquaculture, Viale Piacenza, Lodi, Italy; 3 IGA Technology Service s.r.l., Via Linussio, Udine, Italy; Institute for Biological Research, University of Belgrade, SERBIA

## Abstract

The aim of this study was to evaluate the ability of DNA metabarcoding, by *rbcl* as barcode marker, to identify and classify the small traces of plant DNA isolated from raw milk used to produce Grana Padano (GP) cheese. GP is one of the most popular Italian PDO (Protected Designation of Origin) produced in Italy in accordance with the GP PDO specification rules that define which forage can be used for feeding cows. A total of 42 GP bulk tank milk samples were collected from 14 dairies located in the Grana Padano production area. For the taxonomic classification, a local database with the *rbcL* sequences available in NCBI on September 2020/March 2021 for the Italian flora was generated. A total of 8,399,591 reads were produced with an average of 204,868 per sample (range 37,002–408,724) resulting in 16, 31 and 28 dominant OTUs at family, genus and species level, respectively. The taxonomic analysis of plant species in milk samples identified 7 families, 14 genera and 14 species, the statistical analysis conducted using alpha and beta diversity approaches, did not highlight differences among the investigated samples. However, the milk samples are featured by a high plant variability and the lack of differences at multiple taxonomic levels could be due to the standardisation of the feed rationing, as requested by the GP rules. The results suggest that DNA metabarcoding is a valuable resource to explore plant DNA traces in a complex matrix such as milk.

## Introduction

Current market trends show a growing interest among consumers in the origin of food products [1–3]. In this regard, PDO (Protected Designation of Origin) and PGI (Protected Geographical Indication) products, above others, are able to guarantee authenticity, tradition, taste, typicality, link with the land of origin, safety and traceability [4, 5]. In details, the PDO certification ensures that the product is legally guaranteed by the European Union as authentic, or manufactured in a specific geographical area with the expected ingredients [6, 7]. An accurate control system is included in the certification itself (public supervision, third-party control and self-control). Designations such as PDO and PGI aim to protect the quality standards of agri-food products, safeguard their production methods, provide consumers with clear information on the characteristics that add value to products. This enormous wealth of information for the consumer is ensured by compliance with production specifications [8].

Grana Padano (GP) is one of the most popular Italian PDOs, both in Italy and internationally. In 2020, the GP production reached the quota record of 5,255,443 cheese wheels. With a total production value of 4.5 billion Euros, GP represents 59% of the entire Italian PDO-PGI food production, and 52.6% of PDO-PGI food export [9]. GP is typically produced from raw cow’s milk collected from animals bred in 32 provinces of Northern Italy associated with the Grana Padano Protection Consortium which operates in compliance with the rules defined by the product regulations. As such, the quality of the GP cheese is closely related to the typical climatic and environmental conditions, and forage quality [10, 11].

In support of certified productions, the EU recognizes the importance of food traceability, *i*.*e*. the capacity to track food in all stages of the production chain [12]. Food traceability becomes even more important when the area of production influences the quality of the final product. In this context, recent advances in molecular biology make DNA markers potentially valuable tools to track the raw material in the food chain. In fact, DNA has higher stability compared to other biological markers, such as proteins, and can be isolated even in matrices with extremely low contents [13–15]. In this regard different studies have been described for enhancement of GP or other dairy products: Rocchetti et al. (2018) [16] outlined a metabolomic approach to ensure the authenticity of GP, Faustini et al. (2019) [17] reported on a volatilome study on milk destined to the production of both GP and Parmigiano Reggiano cheeses and, metabarcoding of bacterial communities has been used for the preservation and valorisation of similar food products [18–20]. Several studies have shown that feed-derived plant nuclear and/or chloroplast DNA fragments can also be detected in milk [21]. In fact, small fragments of plant DNA are able to cross the intestinal barrier and enter the bovine bloodstream. However, this DNA is highly fragmented, although it is not possible to establish at what level, as the feed is subjected to the production processes of milling, extrusion, ensiling, grinding, steam heating and pelletizing and, after ingestion, to its elaboration/degradation in the gastrointestinal tract [22, 23]. In these cases, where the DNA is subjected to severe treatments and is present in small quantities, its isolation is particularly difficult and, in the absence of commercial kits optimised for this purpose, it is necessary to implement appropriate molecular approaches based on the analysis of very short DNA fragments. Although some studies were conducted for plant DNA detection in milk using classical approaches [20–24], to the best of our knowledge there are no published data about the detection of residual plant DNA in cow’s milk by High-Throughput DNA Sequencing (HTS) metabarcoding.

Therefore, the aim of this work was to investigate whether chloroplast DNA metabarcoding and dedicated data analysis could be a suitable tool to identify and classify the small traces of plant DNA isolated from bulk raw milk, in order to contribute to development of new traceability systems and to support existing one.

## Materials and methods

### Samples collection

A number of 42 GP bulk tank milk samples of 50 ml volumes were collected from cows fed according to PDO Specification rules [10] in 14 dairies located in the GP cheese production area in the provinces of Verona, Piacenza, Trento, Vicenza, Brescia, Cremona and Cuneo, and stored at -20°C until analysis.

One additional bulk milk sample with a known diet (KD), was also collected in the Lombardy region from a farm of Italian Friesian breed and quickly frozen at -20°C. The animals bred in this farm were fed with feed and forages mainly composed by barley, maize, soy, sunflower, beet, alfalfa, sorghum and sugar cane in different proportion according to the food plan, and water was offered ad libitum.

Three forage samples with known composition were collected, one sample was represented by soy flour (*Glycine max*) and two forages, forage_1 and forage_2 allowed by GP PDO production specification rules compounded with variables portion of maize, wheat, soy, sunflower, sugar beet with different treatments and their by-products.

The KD sample and forages with known composition were collected for permitting us to monitor all steps of the analysis from DNA extraction and amplification through sequence data filtering and provided a very useful way to evaluate the performance and effectiveness of our experimental procedure.

### DNA isolation

All milk samples were thawed at 4°C for 16 hours and total DNA was extracted from 20 ml in phenol/chloroform according to Ponzoni et al. (2009) [21]. As a minor modification of the original method, the first incubation step was conducted at 65°C for 6 hours under constant agitation.

Plant DNA was extracted from forages using the DNeasy Plant Mini Kit (Qiagen, Hilden, Germany) according to the manufacturer’s protocol.

Purity and quantity were determined with Nanodrop Spectrophotometer 2000 (Thermofisher Scientific, Wilmington, DE, USA).

### RuBisCO gene amplicon molecular identification

To confirm the presence of plant nucleic acid in the total DNA extracted from milk, a 351bp RuBisCO gene fragment was amplified with the following primers: RUB F2 TTGGCAGCATTCCGAGTAAC and RUB R2 GTGAGGCGGACCTTGGAAAG [25] by using the GeneAmp PCR System 9700 (Applied Biosystems Foster City, CA). The PCR condition included a denaturation step at 95°C for 15’, followed by 35 cycles of 94°C for 30”, 57°C for 90’ and 72°C for 90’ followed by a final elongation at 72°C for 10’. Fragments were visualised on the Bioanalyzer (Agilent Technologies, Waldbronn, Germany) and only samples with positive amplification were submitted to DNA metabarcoding analysis.

### Library preparation and sequencing

DNA samples were amplified in two steps, as follows: an initial PCR amplification of the variable *rbcL* region of the RuBisCO gene was conducted using locus specific primers with the following Illumina adapter overhangs:

**rbcL F** 5’- TCGTCGGCAGCGTCAGATGTGTATAAGAGACAGATGTCACCACAAACAGAGACTAAAGC-3’ and **rbcL R** 5’- GTCTCGTGGGCTCGGAGATGTGTATAAGAGACAGGTAAAATCAAGTCCACCRCG -3’ and a subsequent amplification that integrates relevant flow-cell binding domains and unique indices (Nextera XT Index Kit, FC‐131‐1001/FC‐131‐1002). Libraries were sequenced on MiSeq instrument (Illumina, San Diego, CA) using the 300 bp paired-end mode.

### Assembly of a local database and metabarcoding analysis

For the taxonomic classification, a local-database of the potential dietary species was created by using 1426 *rbcl* sequences downloaded from NCBI Nucleotide section. Species admitted by the GP PDO Production Specification Rules (clause 4) were also included.

The analysis pipeline was performed as briefly described, as follows: raw reads were trimmed in order to eliminate primer sequences by *cutadapt* (Ref1) with the following parameters:—*anywhere* (on both adapter sequences)—*overlap 5—times 2—minimum-length 35—mask-adapter*. Low-quality bases were removed from 3’ by erne-filter (Ref2) by applying default parameters, excluding reads <60 bp from the further analysis, and reads with an error rate >1% were removed. Chimeric sequences were then removed with an uchime_denovo (Ref3) implemented by usearch (https://www.drive5.com/usearch/), using default parameters.

Reads were clustered to a minimum identity of 97% generating representative sequences (cluster_fast implemented in usearch) and blasted against the local-database with e-value 0.01. At this point, blast hits were analysed and only the lowest unambiguous taxonomy was reported, *i*.*e*. if there are best hits with the same score indicating different lineage, last common ancestor is indicated. The information was then compacted in several Operational Taxonomic Unit (OTU) tables, depending on the considered taxonomic level, either family, genus or species.

### Data filtering

A further filter was applied to each resulting OTU table to remove entries having less than 5 reads and binning them to unassigned (UN). Out of the assigned reads for each sample, entries representing more than 1% of the reads of a sample were labelled as “dominant” [17].

### Statistical analysis

Species richness was investigated through the analysis of rarefaction curves to verify that the reading depth was adequate. The function *rarecurve* from R package *vegan* [26] with parameter *step = 100* was used. Alignment results were filtered for *percent_identity* > 95% and *e-value* < 1e-5. In case of multiple alignments, the one with the smallest *e-value* was retained. A portion of obtained sequences did not align on the *rbcL* database of interest. Clusters not aligning on the *rbcL* database were aligned via blast on the *Bos Taurus* 3.1.1 genome via the NCBI online nucleotide Blast tool (https://blast.ncbi.nlm.nih.gov/).

In this study each sample was considered as an isolated ecosystem, thus allowing the use of statistical tools developed in the ecological field such as alpha (Shannon and Simpson) and beta diversity analysis [27–30]. These two methodological approaches aim to provide objective and comparable measures of biological diversity present in each community. All the considered diversity statistics were computed using the R package *vegan* [26].

## Results

### DNA metabarcoding analysis

The data sets supporting the results of this article are available in the NCBI’s Sequence Read Archive (SRA) repository at the link https://www.ncbi.nlm.nih.gov/bioproject/PRJNA898273.

A total of 700,346 paired-end-mode reads (range 217,739–261,868) with 99% of reads assigned to plant Kingdom were obtained for soy flour, forage_1 and forage_2. Rarefaction curves showed that samples reached stability in the species richness given the amount of available reads (S1 Fig).

Results relatively to forages, KD sample and GP milk samples are shown in Table 1.

**Table 1:** Total number of reads and assigned reads and relative percentages produced in forages and milk samples.

Sample Type	N_sample	Tot_Reads	Tot_Reads_ASS	%Eukaryota	%UN_Eukaryota
Forages	3	700,346	696,264	99.2	0.73
GP milk samples	42	8,399,591	260,784	7.21	92.78
KD sample	1	190,469	39,616	20.8	79.2

Overall, a total of 8,399,591 reads were produced with an average of 204,868 per sample (range 37,002–408,724); more specifically, 1–36.6% of reads were assigned to plant Kingdom.

Reads not assigned to plant Kingdom were aligned to the *Bos taurus* 3.1.1 genome. On average, 63% of them were mapped, confirming the bovine origin.

Only one sample showed the extremely low number of reads equal to 31 and was, therefore, discarded from subsequent analyses.

In the KD sample a total of 190,469 reads were obtained, 21% of reads were assigned to plant Kingdom.

The total sequences obtained were clustered around 4811 centroids and subsequently aligned on the local-database of 1426 *rbcl* sequences. Samples resulted in 16, 31 and 28 dominant OTUs at family, genus and species level, respectively (Table 2).

**Table 2:** Number of raw OTUs, OTUs with reads > 5 and dominant OTUs at family, genus and species level in forages and GP milk samples.

	Forages (n = 3)			GP milk samples (n = 41)		
Taxonomical level	Raw OTUs	Otus reads >5	Dominant OTU	Raw OTUs	Otus reads >5	Dominant OTU
Phylum	1	1	1	1	1	1
Family	11	11	4	10	8	7
Genus	30	27	9	25	16	14
Species	44	34	9	36	16	14

### Taxonomic analysis

**Fig 1:**
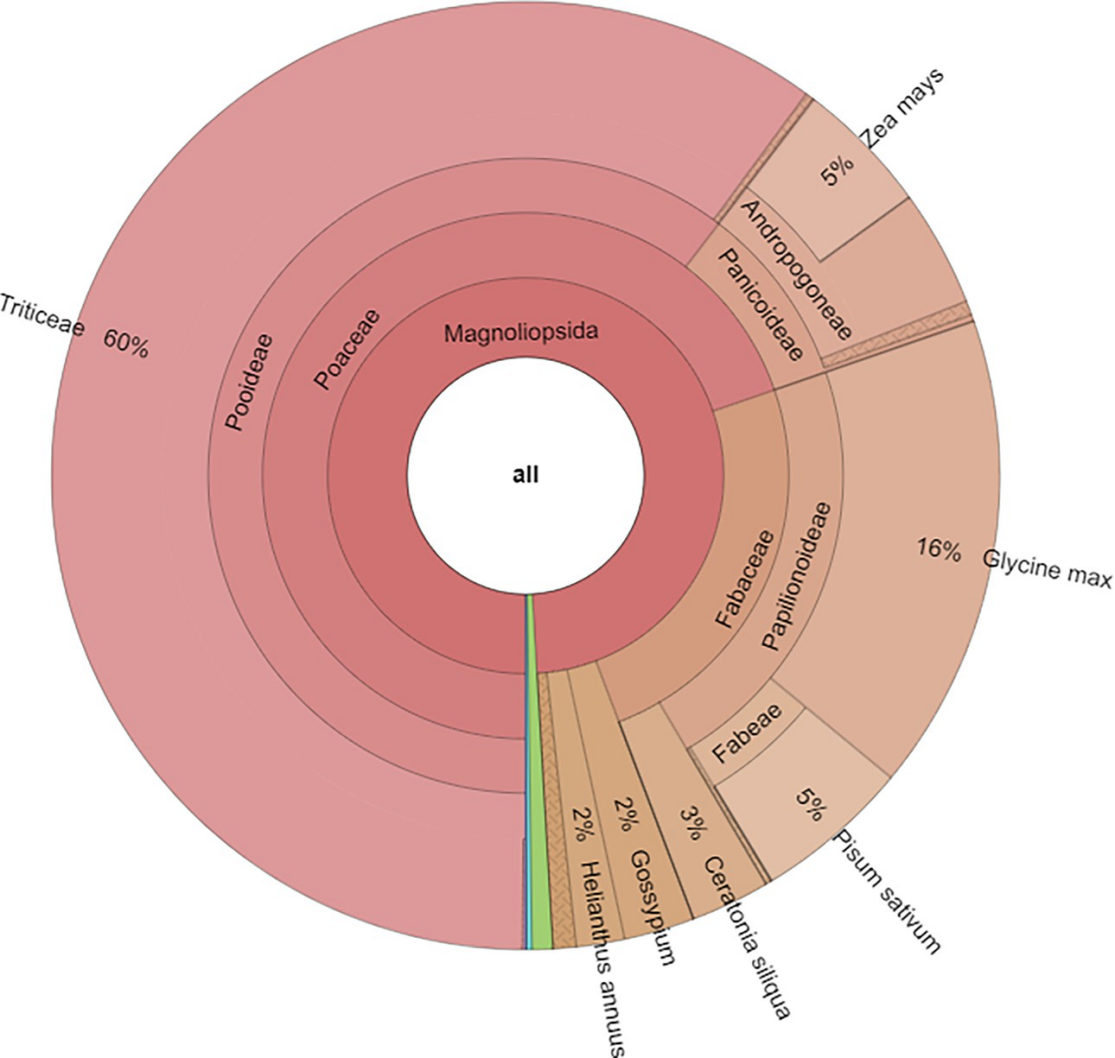
Taxonomic contents of the forage_1.

**Fig 2:**
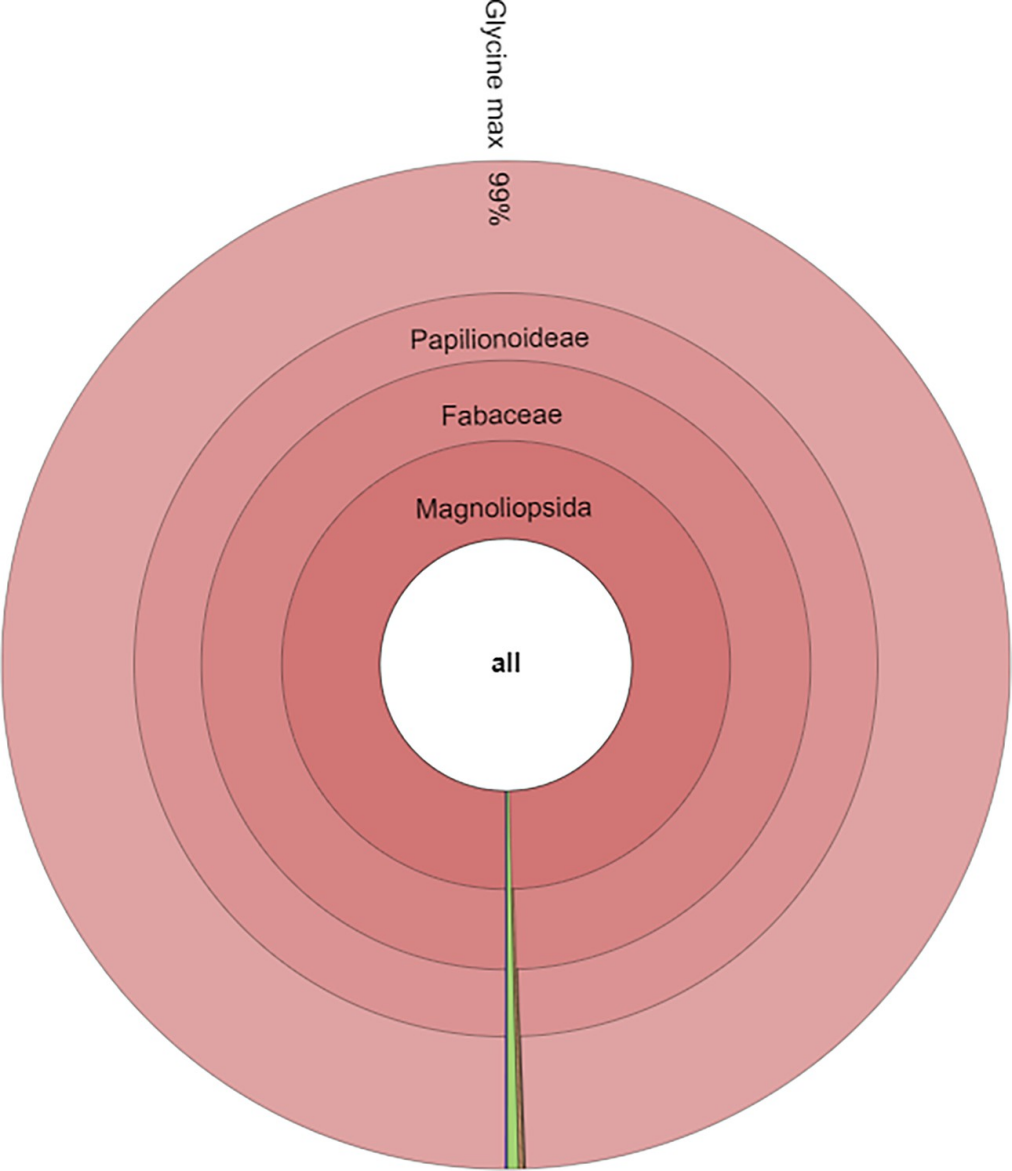
Taxonomic contents of the soy flour.

The taxonomic analysis for the three forage samples showed that the most abundant families in forage_1 and forage_2 were Poaceae (69.3% and 74.8%, respectively) and Fabaceae (24.3% and 17.2%, respectively), while for the soy flour the most represented family was confirmed to be Fabaceae (99.5%). At genera level, in forage_1 the most represented were Glycine (16.28%) followed by Pisum (5.32%), Ceratonia (2.57%), Gossypium (2.18%), Helianthus (1.64%) and Zea (1.54%). In forage_2, the percentage of Glycine was equal to 14.38% followed by Helianthus (2.39%), Gossypium (2.36%), Ceratonia (1.68%), Pisum (1.02%) and Zea (0.87%). Regarding soy flour sample, Glycine was equal to 99.39% with minimal percentages of other genera. At species level, for forage_1 and forage_2 the most abundant specie was Glycine max (16.02% and 14.26%, respectively) followed by Pisum sativum (5.3% and 1.02%, respectively), Ceratonia siliqua (2.5% and 1.68%, respectively), Helianthus annuus (1.64% and 2.39%, respectively) and Zea mays (1.54% and 0.88%, respectively). Graphical representations of taxonomic analysis for one forage and soy flour were reported in Figs 1 and 2.

In GP milk samples, the metabarcoding analysis associated all the assigned reads to the Streptophyta Phylum. Analysis of deeper taxa identified 7 families (*Fabaceae*, *Convolvulaceae*, *Poaceae*, *Rubiaceae*, *Malvaceae*, *Asteraceae and Ranunculaceae*), 14 genera (*Ceratonia*, *Cuscuta*, *Glycine*, *Triticum*, *Gallium*, *Gossypium*, *Medicago*, *Panicum*, *Lolium*, *Helianthus*, *Pisum*, *Zea*, *Delphinium and Vicia*) and 14 species (*Ceratonia siliqua*, *Triticum aestivum*, *Medicago sativa*, *Gossypium mustelinum*, *Glycine max*, *Panicum pygmaeum*, *Panicum wiehei*, *Lolium perenne*, *Pisum sativum*, *Helianthus annuus*, *Vicia faba*, *Zea mays*, *Delphinium grandiflorum and Secale cereale*). Among the seven families identified, the most abundant were: Fabaceae (55.9%), Convolvulaceae (26.6%) and Poaceae (9.4%), followed by Rubiaceae (4.3%), Malvaceae (3.4%), Asteraceae (0.2%) and Ranunculaceae (0.1%). At a lower taxonomic level, the most representative genera were *Ceratonia* (33.9%), *Cuscuta* (26.9%), *Glycine* (21.2%) and *Triticum* (5.8%).

**Fig 3:**
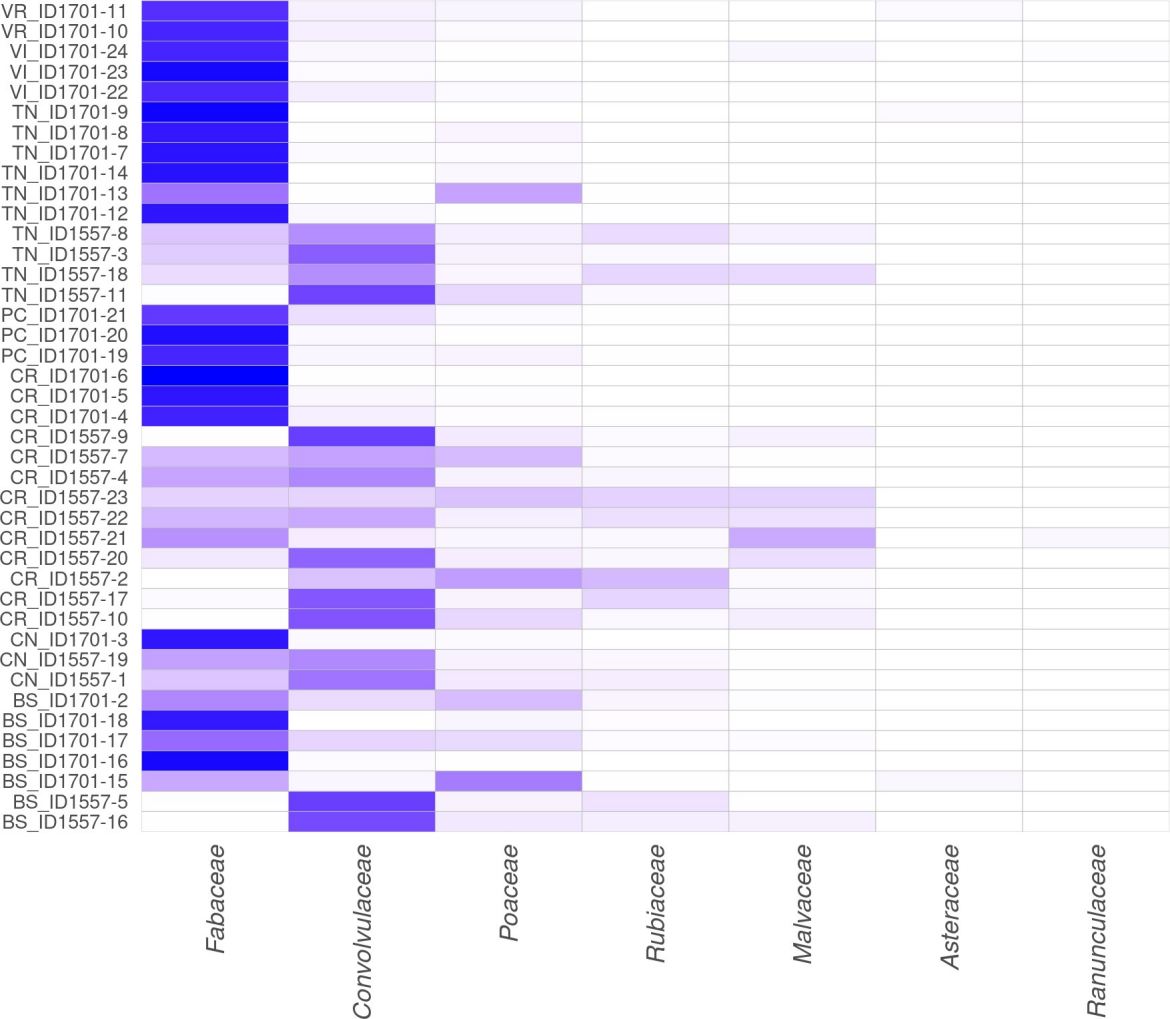
Abundance detected in GP milk samples at family taxonomic level. Heatmap showing the abundance of the seven dominant families (≥1% total reads on at least one sample) found in milk samples. The colour scale indicates the species’ relative abundance: more intense is the colour, more abundant are the species. The province of origin is indicated by the first two letters of each sample.

**Fig 4:**
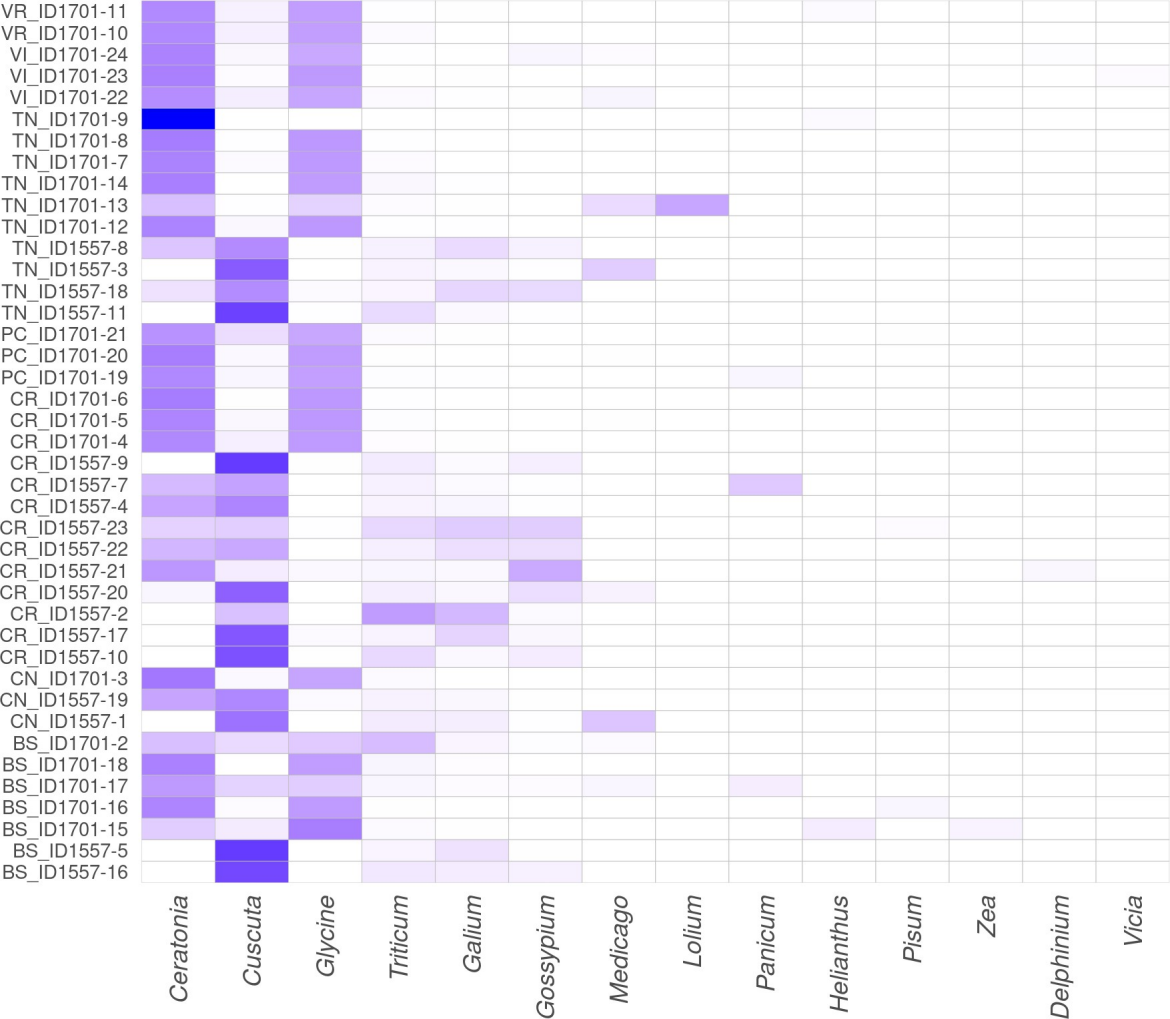
Abundance detected in GP milk samples at genus taxonomic level. Heatmap showing the abundance of the fourteen dominant genera (≥1% total reads on at least one sample) found in milk samples. The colour scale indicates the species’ relative abundance: more intense is the colour, more abundant are the species. The province of origin is indicated by the first two letters of each sample.

**Fig 5:**
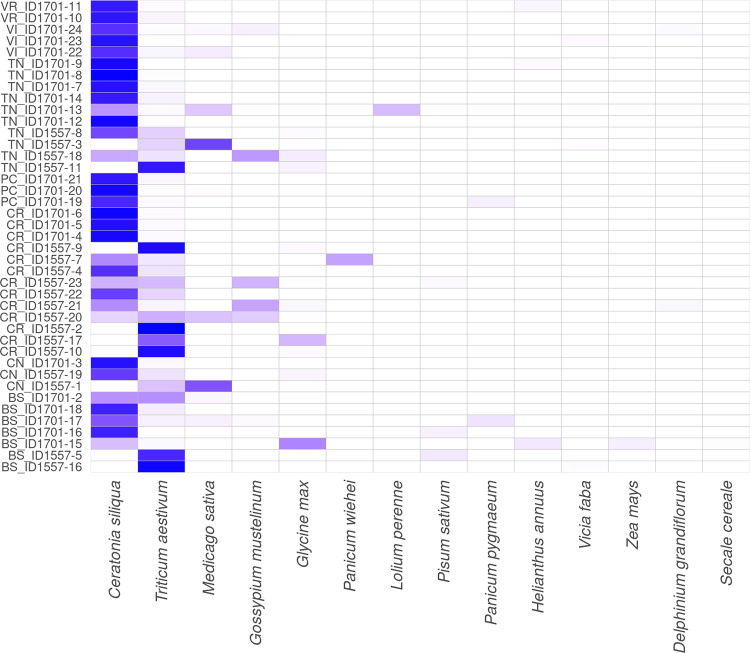
Abundance detected in GP milk samples at species taxonomic level. Heatmap showing the abundance of the fourteen dominant species (≥1% total reads on at least one sample) found in milk samples. The colour scale indicates the species’ relative abundance: more intense is the colour, more abundant are the species. The province of origin is indicated by the first two letters of each sample.

With regard to the abundance in Figs 3–5, are shown the heatmaps for dominant (>1%) centroids at different taxonomic levels: family, genus and species, respectively.

**Fig 6:**
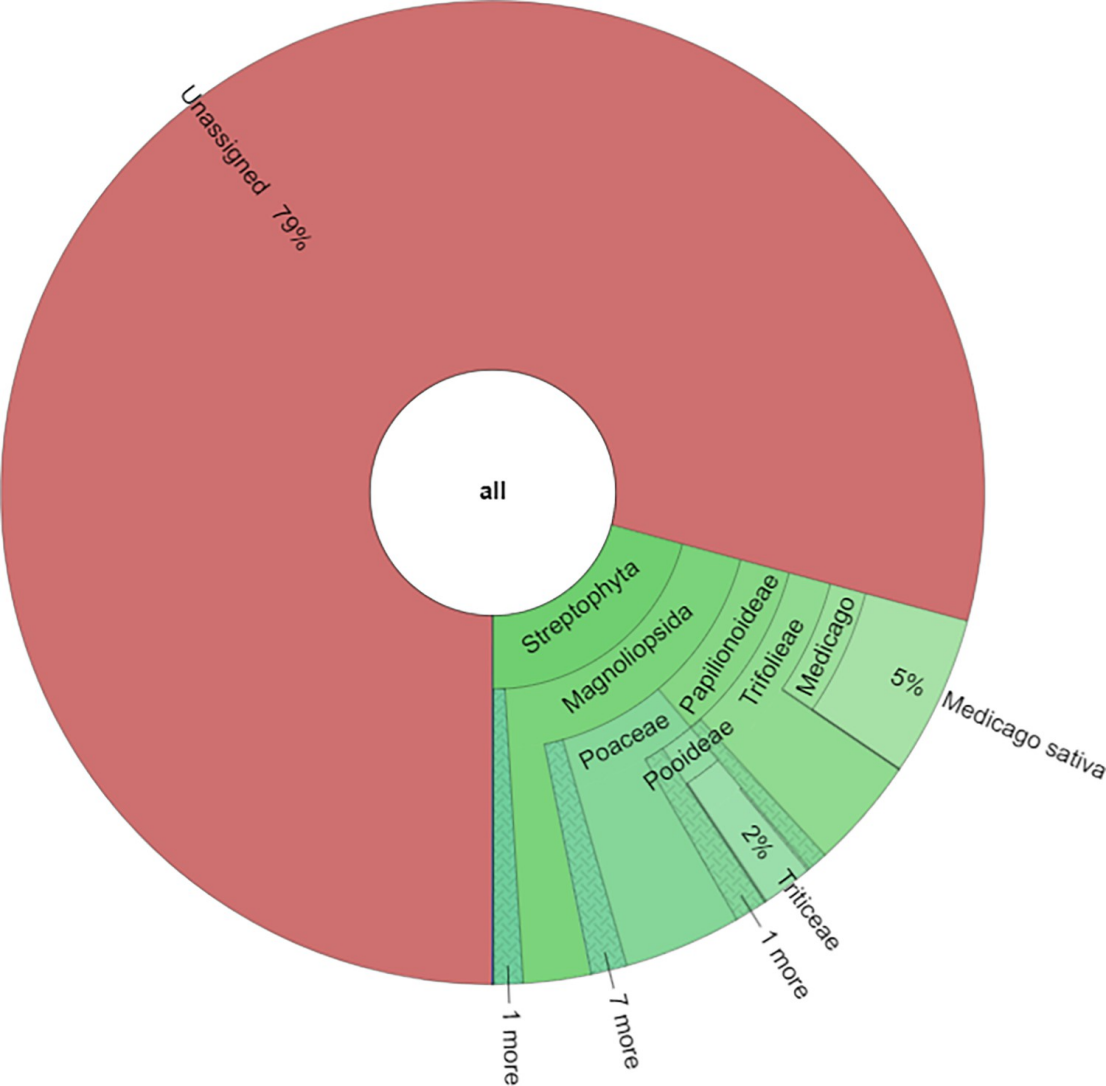
Taxonomic contents of the KD sample.

In the KD sample, the metabarcoding analysis associated all the assigned reads to the Streptophyta Phylum, identifying 10 families, of which the more abundant are Fabaceae (46.44%), Poaceae (34.43%) and followed by Asteraceae (4.60%). At a lower taxonomic level, the most representative genera were *Medicago* (25.5%), *Zea* (5.11%), *Picea* (4.52%) and *Glycine* (3.14%). At species level the most abundant species were *Medicago sativa* (25.51%), *Zea mays* (5.11%) and *Glycine max* (3.14%), shown in Fig 6.

### Statistical analysis

**Fig 7:**
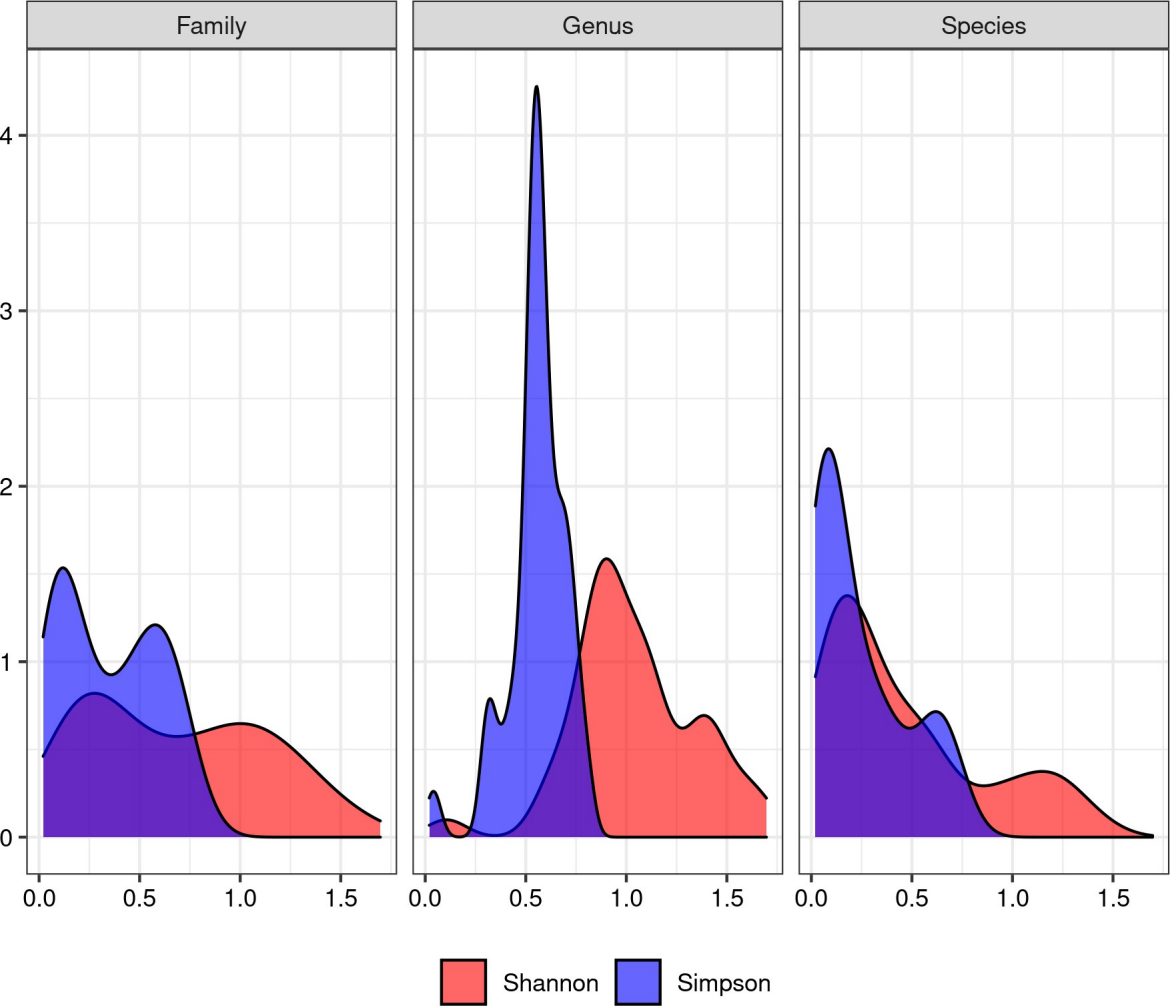
Distribution of alpha diversity computed using Shannon’s and Simpson’s indexes. The three boxes report indexes computed at different taxonomic levels.

Alpha diversity analysis was performed using the “Shannon-Wiener” and “Simpson” diversity indexes. We considered three taxonomical levels, namely family, genus and species. Each sample was considered as an isolated ecosystem. Index distributions are reported in Fig 7 and averaged in Table 3.

**Table 3:** Average values for Shannon and Simpson richness indexes for all samples, computed at three different taxa levels. Significance test p-values computed via ANOVA grouping the samples by province.

Richness index		Taxa level		
		Family	Genera	Species
Shannon	Average	0.684	1.038	0.46
	p-value	0.108	0.128	0.322
Simpson	Average	0.350	0.557	0.245
	p-value	0.083	0.267	0.285

**Fig 8:**
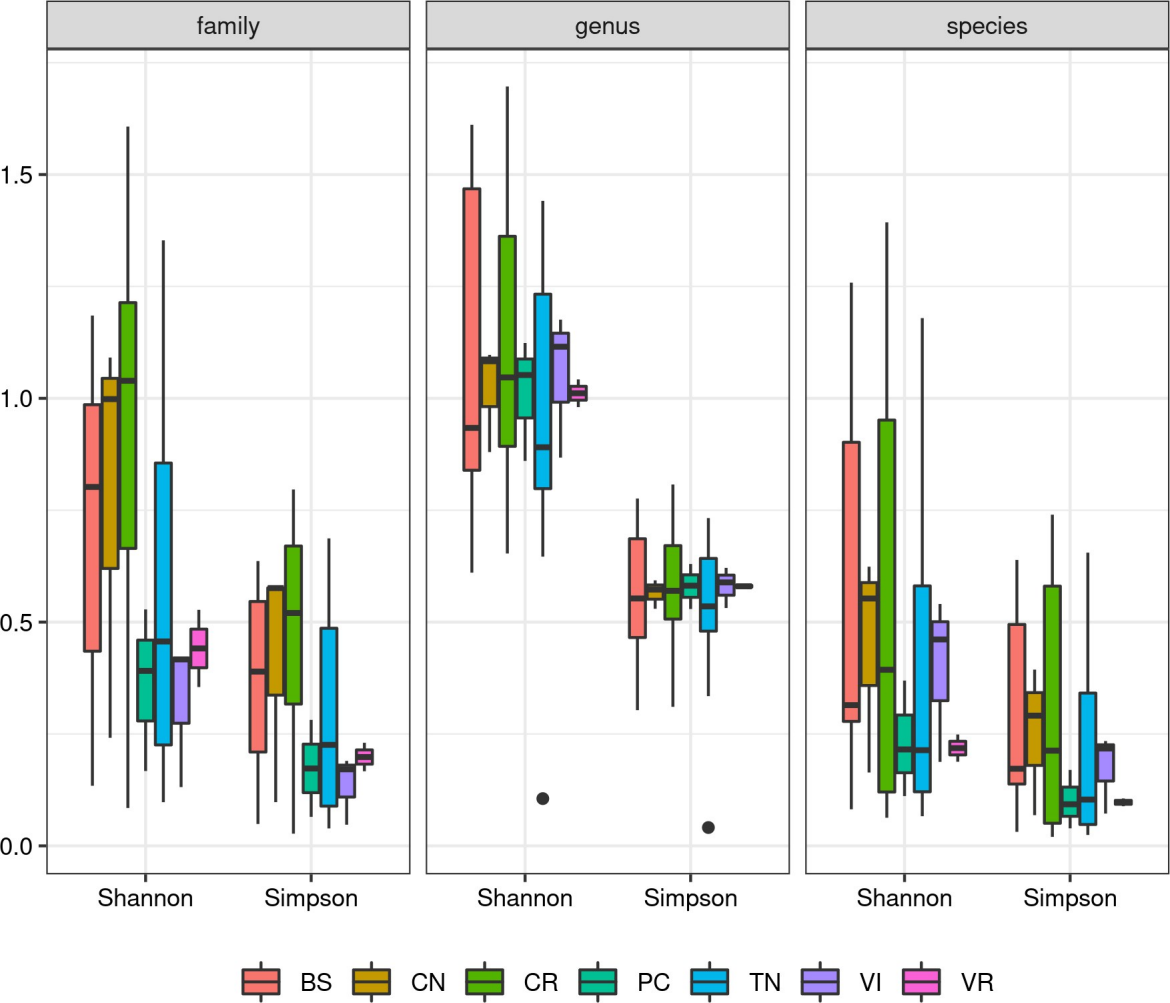
Distribution of alpha diversity computed using Shannon’s and Simpson’s indexes. Colours indicate different provinces of origin. The three boxes report indexes computed at different taxonomic levels.

Samples can also be grouped by province of origin, thus allowing investigate differences between groups. Distributions of diversity indexes are reported in Fig 8. Moreover, we performed a standard ANOVA to formally ascertain the presence of statistically significant differences between provinces. Resulting p-values are also reported in Table 3.

Results revealed no statistically significant differences for both indexes at three taxonomic levels (family, genus and species), with all p-values ≥ 0.05. The Shannon index shows a clearly non-normal distribution (Fig 7). In fact, it appears to be bimodal, *i*.*e*. as if it was the union of two distributions, one with lower and one with higher average diversity.

**Fig 9:**
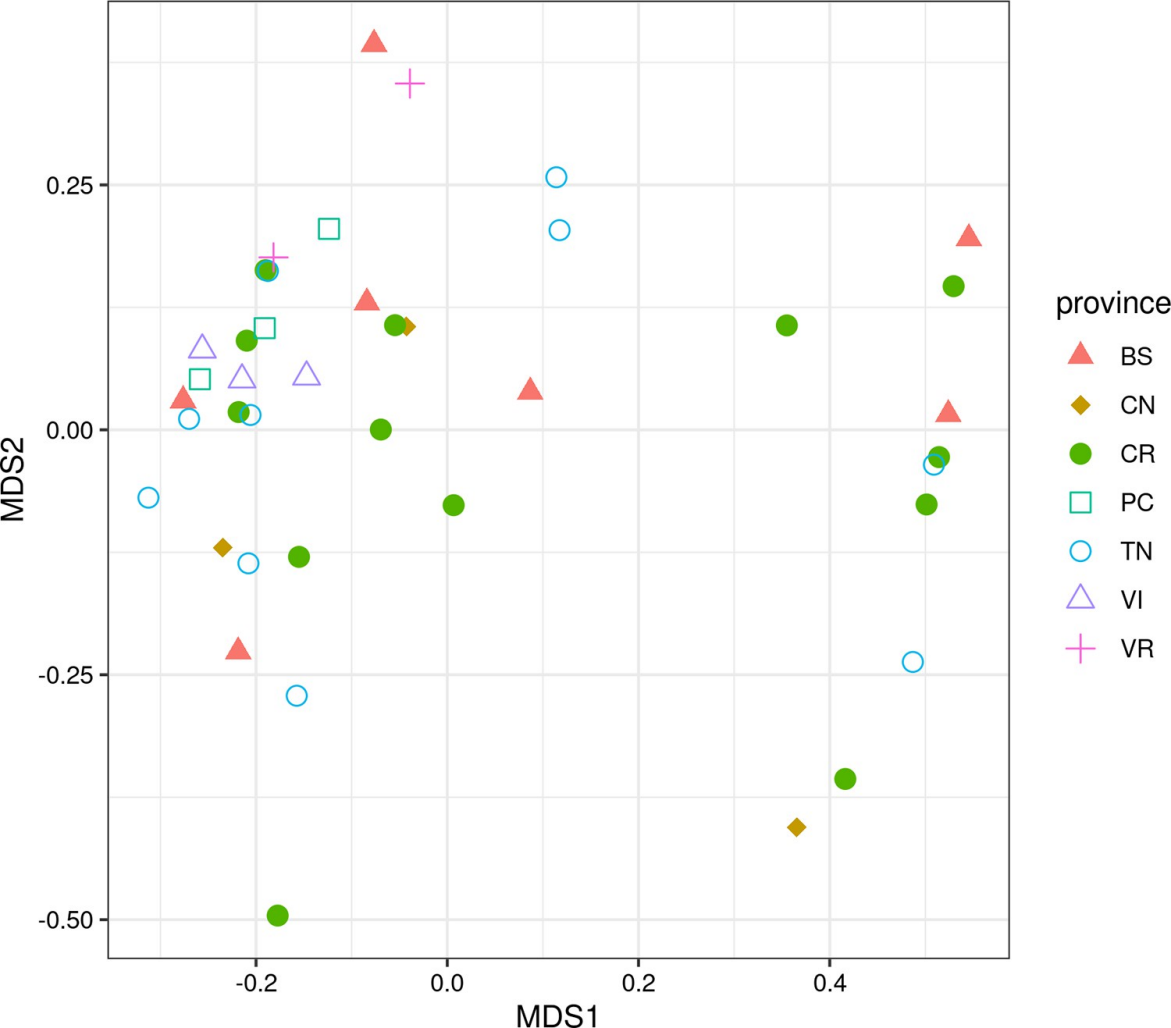
Diversity analysis—beta diversity. Beta diversity computed using Bray–Curtis index and then transformed via Multi-Dimensional Scaling for milk samples by province.

Beta diversity also considers information about biological richness, but measures the variation between pairs of different communities using the Bray-Curtis index. Once the (square, symmetrical) diversity matrix is obtained we applied Multi Dimensional Scaling (MDS) to allow data exploration. Results are reported in Fig 9.

## Discussion

In this work, bulk milk samples collected within the GP production area were investigated by a metabarcoding approach in order to identify and classify the residual plant DNA content. As far as we know, there are no published studies on DNA metabarcoding for the detection of residual plant DNA in bulk cow’s milk. In this regard, only a few studies have been conducted with the same goal using classic PCR approaches [21], while others have used different biological matrices such as faeces, intestinal contents and honey [31, 32]. One of the difficulties in characterising plant DNA in milk compared to other biological matrices derives from the fact that plant components resulting from the diet are strongly degraded and fragmented due to their passage through the digestive system and because of technological processes for feed production. At the same time, it is important that the chloroplast DNA (cpDNA) target can be extracted with the least amount of nuclear and mitochondrial DNA and other contaminants, such as polysaccharides and resins, that can interfere and reduce the efficiency of the subsequent applications [33].

With particular reference to these aspects, the extraction method adopted in the present work proved to be effective for recovering chloroplast DNA from plants in milk. Furthermore, of considerable importance for the subsequent development of the method, the Consortium for the Barcode Of Life (CBOL) Plant Working group identified plastid coding region *rbcl and matK as* core-barcode for plant identification [34]. In our present work the *rbcl* marker was chosen because it is the most characterised plastid coding region in the GenBank database and for its higher universality compared to other DNA barcode markers described in the literature [35]. Although *matk* is considered a good marker of DNA due to its high-resolution rate, it was excluded since it is difficult to amplify with a single primer pair, especially when compared with *rbcl* which is less problematic not only in amplification [14], but also in sequencing and bioinformatics analysis.

The taxonomic analysis of plant DNA isolated in all the samples was performed with a local database specifically created based on the Italian plant taxa. In fact, as demonstrated by several authors [32, 34, 36, 37], the use of local dedicated databases for the taxonomic assignment reduces the possibility of wrong identifications compared to existing databases that contain millions of reference DNA sequences [38]. Results of the present work proved that DNA metabarcoding was effective to characterise the plant component and to measure the abundance of sequences at phylum, family and genera level in GP milk, in KD sample and in the 3 control forage samples with known composition, allowed the correct identification of the related taxa. Two plants were also detected, *Ceratonia siliqua* at the species and *Cuscuta* at the genus level for which a more in depth classification was not possible; their presence could be due to several factors. At first, DNA metabarcoding can produce different proportion of reads which are not related to the real quantities of each species in the analysed sample [37]. Furthermore, the sequence abundance can also be affected by the density of chloroplasts in different species [39]. Another reason could be the bias of amplification efficiency in PCR towards different species and loci because of the high level of plant DNA fragmentation, that could bring to a sharing of sequences between relative species [40]. The last hypothesis could be referred to an environmental contamination: there are several important sources of terrestrial eDNA that can be traced such as soil, water sources, air and pollen.

The statistical analysis conducted using the alpha approach confirmed the absence of differences among samples collected from the various provinces of the GP production area.

The beta analysis highlighted a subdivision of the samples into two distinct clusters showing how, in some cases, the milk of a specific area could actually belong to both clusters as a further evidence of the high homogeneity among samples. These results are in line with the expected high level of standardization of feed rationing required by the GP standard to ensure the product quality.

Overall, the results suggest that DNA metabarcoding is a valuable resource to explore plant DNA traces in a complex matrix such as milk, those representing a good starting point to develop a new molecular approach or to improve existing ones, in order to add more information to the food traceability.

## Supporting information

S1 Fig Rarefaction curves. (TIF)
