# Supplementary figures and images for: Assessment of residual plant DNA in bulk milk for Grana Padano PDO production by a metabarcoding approach
Source: PLoS One. 2023 Jul 25;18(7):e0289108. doi: 10.1371/journal.pone.0289108 (PMC10368264; doi:10.1371/journal.pone.0289108)

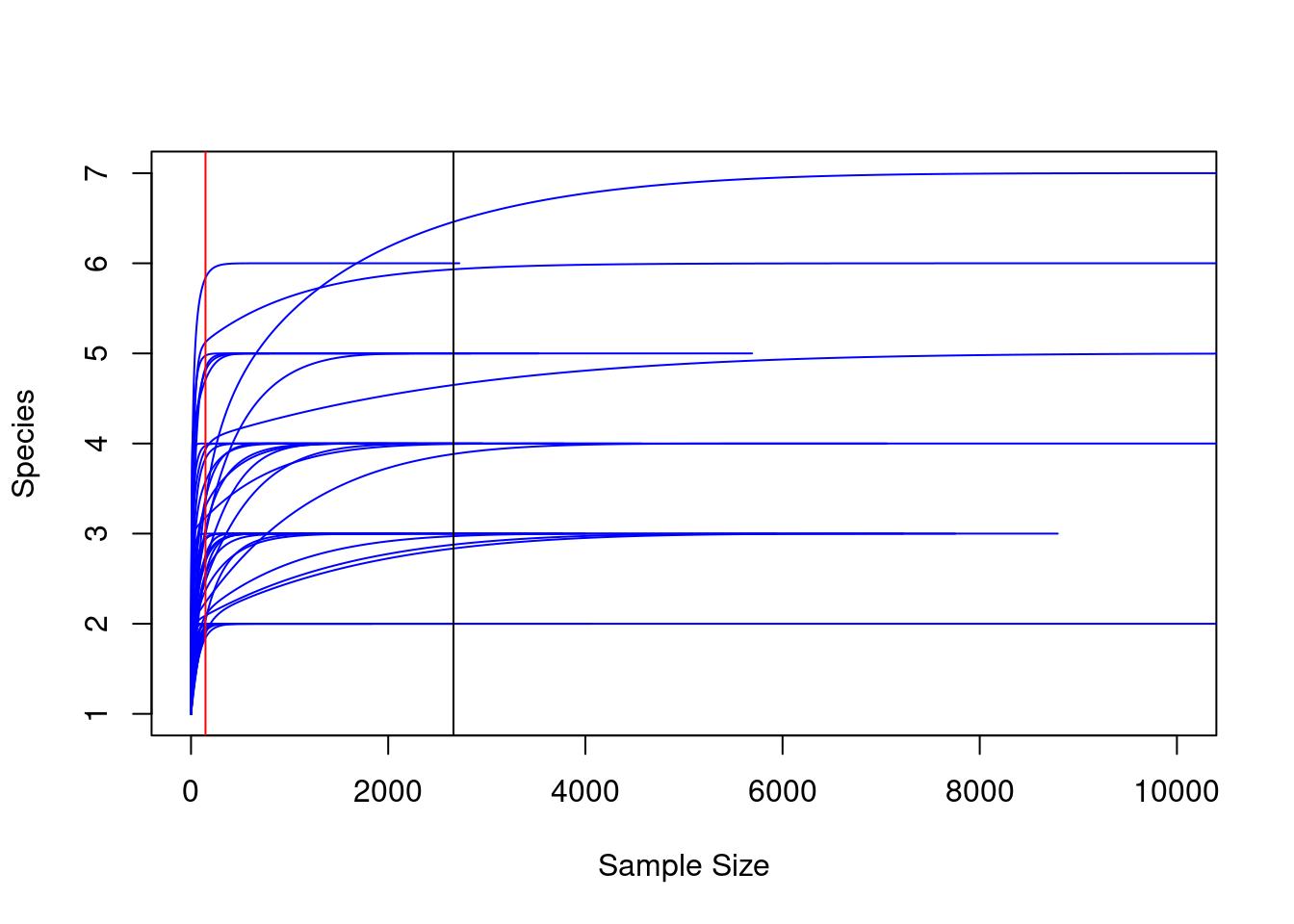

Supplement: S1 Fig — (TIF) [file pone.0289108.s001.tif]
